# Talking to the senses: modulation of tactile extinction through hypnotic suggestion

**DOI:** 10.3389/fnhum.2012.00210

**Published:** 2012-07-17

**Authors:** Angelo Maravita, Mario Cigada, Lucio Posteraro

**Affiliations:** ^1^Department of Psychology, University of Milano-BicoccaMilano, Italy; ^2^Riabilitazione SpecialisticaOspedale di Suzzara SpA (MN), Italy

**Keywords:** hypnosis, neuropsychology, brain-damage, tactile extinction, rehabilitation

## Abstract

Following brain damage, typically of the right hemisphere, patients can show reduced awareness of sensory events occurring in the space contralateral to the brain damage. The present work shows that a hypnotic suggestion can temporarily reduce tactile extinction to double bilateral stimulation, i.e., a loss of contralesional stimuli when these are presented together with ipsilesional ones. Patient EB showed an improved detection of contralesional targets after a single 20-min hypnosis session, during which specific suggestions were delivered with the aim of increasing her insight into somatosensory perception on both sides of the body. Simple overt attention orienting toward the contralesional side, or a hypnotic induction procedure not accompanied by specifically aimed suggestions, were not effective in modulating extinction. The present result is the first systematic evidence that hypnosis can temporarily improve a neuropsychological condition, namely Extinction, and may open the way for the use of this technique as a fruitful rehabilitative tool for brain-damaged patients affected by neuropsychological deficits.

## Introduction

Brain damage can deeply alter conscious perception of sensory events. A striking example of this is extinction to double simultaneous stimulation, whereby patients can detect unilateral stimuli on both sides of space, but fail to detect stimuli delivered to the side contralateral to the damaged hemisphere [more often left-sided stimuli following a right hemispheric lesion (Vallar et al., 1994a)], when these are delivered together with simultaneous ipsilesional ones (Bender, 1945; Bender and Diamond, 1975; Vallar and Maravita, 2009). Extinction is believed to originate from an abnormal competition for attentional resources between stimuli on opposite sides, which would cause the loss of the contralesional, “weaker,” stimulus on bilateral presentations (Driver et al., 1997). A striking aspect of extinction is that, by definition, the deficit cannot be fully explained by a primary sensory loss (although this often co-exists), as shown by the correct detection of unilateral contralesional stimuli and by the evidence for various degree of implicit processing of the unreported targets (Maravita, 1997; Berti et al., 1999). The core deficit of these patients, therefore, would be at a higher level of perceptual processing, where sensory stimuli escape conscious detection after a certain degree of sensory analysis.

Extinction usually accompanies, or follows the more complex syndrome of Unilateral Spatial Neglect (USN), although it is dissociable from it (Cocchini et al., 1999). USN a complex, multicomponential syndrome in which patients fail to report bodily or extrapersonal stimuli delivered to the side contralateral to the damaged hemisphere (typically left-sided stimuli following a right hemispheric lesion), even if delivered in isolation (Heilman et al., 2003; Husain, 2008; Vallar and Maravita, 2009).

A clinically relevant feature of USN and extinction is that patients are typically unaware of their neuropsychological deficit (as well as of other co-existing primary sensory and motor deficits) a condition named anosognosia (Vallar and Ronchi, 2006). USN (Di Monaco et al., 2011), anosognosia (Gialanella et al., 2005) and to some extent even extinction (Rose et al., 1994) are regarded as negative predictors of outcome in brain-damaged patients with sensorimotor deficits. In particular, anosognosia seems to have a pervasive negative influence on the functional recovery after stroke (Gialanella et al., 2005) since it significantly limits the active participation of patients to any rehabilitation training. For this reason there is a strong need for a better understanding of these deficits and to find novel approaches by which residual sensory processing can be made available to awareness, even in patients who do not fully acknowledge their deficits and, therefore, are not likely to adopt explicit strategies to improve. Following these principles, many approaches have been proposed to date, such as vestibular stimulation (Cappa et al., 1987), neck muscle vibration (Karnath et al., 1993), TMS (Fierro et al., 2006), prism adaptation (Rossetti et al., 1998; Maravita et al., 2003), and limb crossing (Smania and Aglioti, 1995). These techniques have proved effective in producing short- or long-lasting improvements of USN and extinction in a “bottom up” fashion, i.e., without requiring the patient to adopt any explicit strategy.

In the present work hypnotic suggestion was used as a novel approach to modulate sensory extinction, and in particular tactile extinction. This technique is nowadays regarded as an important tool for studying sensory and cognitive brain functions (Oakley and Halligan, 2009; Raz, 2011) given its efficacy in modulating several electrophysiological and neurofunctional indexes (Rainville et al., 1997; De Pascalis, 1999; Maquet et al., 1999; Cojan et al., 2009), perceptual-cognitive effects (Szechtman et al., 1998; Kosslyn et al., 2000; Raz et al., 2007; Cohen Kadosh et al., 2009; Cojan et al., 2009; Casiglia et al., 2010; Priftis et al., 2011), clinical conditions (Oakley et al., 2002) or even simulating neurological or psychiatric conditions (Halligan et al., 2000; Blakemore et al., 2003).

During hypnosis, participants experience a peculiar condition (also indicated as “state of trance”), which is induced by the hypnotist through specific verbalizations. The nature of the “trance” state is still a matter of discussion. Researchers have found different physiological markers which would characterize the patient in hypnosis, including specific patterns of functional brain activations and deactivations (Maquet et al., 1999), changes in electrical cortical activity (De Pascalis and Penna, 1990) or increased activity in the anterior areas of the “default mode” network in highly susceptible individuals (McGeown et al., 2009). In the clinical practice, the hypnotic state is believed to help the patients accepting suggestions oriented at modifying their behavior not only while in hypnosis but, critically, even once the trance state is over (Erickson, 1980). In the present work we applied (although non-rigidly, as shown in the Methods section) a mainly indirect approach for delivering hypnotic suggestions, in line with the theoretical view outlined by Milton Erikson and currently used by many clinical psychotherapists (Erickson, 1980). This approach typically avoids proposing directive suggestions or the adoption of explicit behavior to the patient, but makes use of metaphoric statements or verbal descriptions that help patients building up insight into their deficit and stimulate the discovery of their own strategies for recovery (Williams, 1983; Matthews, 2000; Maudoux et al., 2007; Ross et al., 2007). Using this approach in hypnosis seemed an ideal way to improve neuropsychological deficits that typically escape the patient's awareness, such as extinction. In the present study we specifically targeted tactile extinction, with the aim of testing whether this deficit could be positively modulated by a single session of hypnosis in a right brain-damaged patient. The rationale of the hypnotic suggestions was that of guiding the patient towards a reduction of competition between the ipsilesional and the contralesional stimuli, following the idea of limited attentional capacity in these patients (Driver et al., 1997). While the rationale of this hypnotic approach was decided before meeting the patient, the details of the suggestions and the specific verbalization useful to reach that aim were decided online, following the imaginative contents produced by the patient during hypnosis.

## Case description

**Figure 1:**
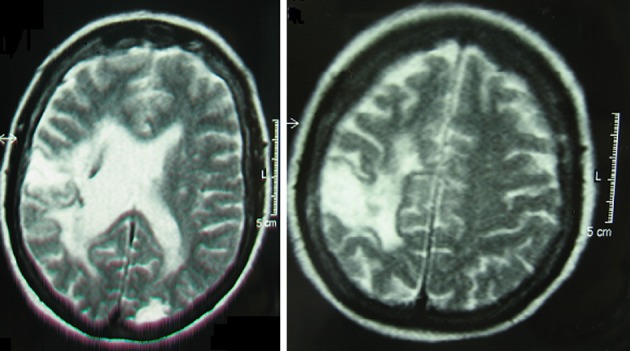
**Magnetic Resonance images of EB's brain lesion**. Following the radiological convention, the right hemisphere is on the left side of the image.

EB is a lively and cooperative 58 year-old, right-handed woman. Two years previous to testing she suffered a large ischemic stroke involving the insular, frontal and temporal regions of the right hemisphere (see Figure 1). Sensory and motor functions were briefly assessed through the standardized neurological examination by Bisiach et al. (1986). With this test both functions are examined according to a three-point scale ranging from score 0 (absence of deficit) to score 3 (maximum deficit). At the time of testing EB presented with left spastic hemiparesis (score 2 for the lower and 3 for the upper limb) and left hemianopia (score 3 for both quadrants). She also presented with USN and tactile extinction, as described below.

### Assessment of tactile sensitivity and USN

Tactile sensation and extinction were tested in the baseline assessment and in the experimental evaluations by computer-controlled devices (Foreman & Co., UK) producing single silent and invisible touches of 100 ms duration. The stimulators were taped on the distal pad of the forefinger of each hand while EB rested the hands on her lap and fixated a central mark placed on the table in front of her. Each testing session comprised the following stimulus types: 12 unilateral left, 12 unilateral right and 24 bilateral, plus 12 catch trials (no stimulation), for a total of 60 trials, delivered in random order within a single session. After each trial the examiner verbally prompted the patient to report the presence and the side of any perceived stimulus, recorded EB's response and started the following trial.

USN was formally tested in the baseline assessment and the experimental sessions, through common standardized tests, namely letter cancellation (Diller and Weinberg, 1977), star cancellation (Wilson et al., 1987), figure and shape copying test (Wilson et al., 1987), and sentence reading test (Pizzamiglio et al., 1990)^1^.

## Methods

### Experimental design

The patient was tested in accordance with the guidelines of the Declaration of Helsinki, in eight sessions distributed over three days during a three-week period. Although the main focus of the hypnotic intervention was extinction, in all sessions EB was tested for tactile extinction and USN, apart from the last session when she was tested for extinction only. The patient was informed of the experimental procedure and that we planned to use hypnosis in order to improve her condition, in particular her perceptual functions, without explaining in full details the experimental design, in order not to influence her performance in the testing sessions. A full debriefing was given to EB at the end of the last experimental session.

The first day (day 1) comprised a first baseline testing of USN and extinction, plus an identical delayed baseline, that was performed after a 20-min interval occupied by an unstructured interview. The delayed baseline was performed in order to check for test–retest reliability of extinction and USN in a sequence comparable to that of the critical experimental sessions with hypnosis (see below). Furthermore, a third session was performed after EB was instructed to voluntarily orient her attention toward the left hand. This manipulation was introduced in order to compare the effect of a simple explicit attention-orienting strategy with the critical hypnosis suggestion. On the second day of testing (14 days after day 1), EB was tested before and after a preliminary hypnotic training (named Hypnosis-1) where she could familiarize with the induction of hypnosis, but without receiving any specific suggestion targeting extinction. On the third testing day (seven days after day 2) two further evaluations were performed, separated by the critical hypnotic induction (Hypnosis-2) specifically aimed at improving extinction, plus a further follow-up testing (Post-hypnosis-2 delayed), performed after 45 min in order to test for any delayed effect.

The number of correct responses in the different conditions was compared using planned Chi-squared tests.

### Description of the hypnotic inductions

#### First induction (hypnosis-1)

This first hypnosis session had the following aims: collecting the clinical history and establishing a good therapeutic rapport with the patient and explaining to EB the principles of hypnosis, with explicit reference to this technique, and the aims of the procedure. Furthermore, in this experimental session the hypnotic induction was performed without any specific suggestion directed at influencing EB's sensory processing, but only as a training for reaching a good state of hypnosis and as a control for any unspecific effect of the hypnotic state on extinction.

A simple progressive relaxation technique, i.e., backward counting, was used by the hypnotist (one of the authors, Mario Cigada). This was meant to facilitate the hypnotic induction, also given that EB appeared very distractible and manifested some difficulty in paying attention to the hypnotist's voice. After some relaxation was obtained, the hypnotist asked the patient to imagine herself in a pleasant place and describe what she imagined. EB referred that it was difficult for her to try and visualize anything, and her level of relaxation was waxing and waning for some time, with frequent interruptions of the hypnotic induction procedure, in which the patient started talking to the hypnotist about her clinical condition. The relaxation procedure was repeated a few times, since EB appeared more focused on the words of the hypnotist. At this stage, EB was able to relax quite easily and spontaneously recollected a trip to Athens occurred 30 years before, describing many images and details. The patient appeared to reach a good level of trance, as suggested by some overt behavioral signs, such as spontaneous eye closing, reduced loudness and tone of the voice, reduced facial expressions, absence of unrequested voluntary movements, decreased breath frequency and muscular relaxation that included the upper and lower limb on the plegic side^2^. In particular we observed a visible reduction of spasticity on the left hand as a consequence of muscular relaxation. This was of much surprise for the patient's daughter, who was present during the hypnosis session, since a reduction of the left hand spasticity was usually very difficult to obtain. After about 15 min, the hypnosis was terminated and the patient showed a sudden change of trunk posture and successive postural adjustments on the wheelchair, eye opening and gaze re-orienting, all suggesting that she was out of the hypnotic state.

#### Second induction (hypnosis-2)

The aim of the second experimental session was to influence the contralesional tactile extinction by specific suggestions during the hypnotic state.

The hypnotist started by using the same progressive relaxation technique (backward counting) used in the previous hypnotic session, in order to reach a state of concentration and relaxation. Again the procedure was initially quite effortful for the patient and required a few minutes to be effective. The trance was progressively deepened by the recall of several pleasant sensations experienced spontaneously by the patient in the previous session. After EB seemed to have reached a trance state, as suggested by similar behavioral signs of as those described above (including, again, visible muscular relaxation with reduced spasticity), the patient spontaneously recalled a car trip to the seaside. EB described the landscape that she imagined to see through the car window. A suggestion was then given that “the windscreen is misted over and that she can try and make left-sided images brighter by cleaning it.” After a while the imaginary trip took EB over a bridge on the Po River (located in the North of Italy). The patient became quite emotional at this memory and her level of concentration decreased. After EB was again relaxed, she was invited to “imagine to dip both hands into the river's fresh water and try and experience how pleasant is to play with the sensations coming from both hands, for example, by reducing the sensitivity of the right hand and increasing that of the left hand.” The patient found this exercise pleasant and easy to perform. The sensory manipulation was suggested a few times and it was also suggested that she “could keeping on playing with the sensitivity from the hands as if she was performing a physical training, in order to get stronger and feeling better, and that she could learn this pleasant exercise and repeat it from time to time in her daily life.” The rationale of this statement was to favor EB's insight into tactile experience from both hands, by suggesting a positive association between somatosensory imagery and physical health, with the final aim of an improvement of extinction. The suggestion that this exercise could be repeated in the future as a sort of healthy training was aimed at anchoring EB's experience in hypnosis with her daily life, in order to help her favoring a process of self-healing.

The patient suddenly started crying, while recollecting her mother, who died a few years before and who joined the patient during that trip to the river. After she felt calmer, EB's somatosensory imagery was again stimulated by inviting the patient to “visualize her body walking around, and to concentrate on the somatosensory sensations coming from the movement of the left and right sides of the body.” After EB has concentrated on this task for a while, describing sensation of touch, pressure, and temperature coming from her body, she was asked to slowly end her imaginary, and hypnosis was terminated. Again EB showed clear signs of sudden, spontaneous postural and attentional re-orienting. This session of hypnosis lasted around 20 min.

## Results

### Baseline testing of extinction and USN (Tables 1, 2)

When tested for tactile extinction EB showed perfect detection of right-handed stimuli, reduced sensitivity on the left-handed stimuli (8 out of 12 correctly perceived) and 100% extinction of the left touches on bilateral stimulations (Table 1). There were only occasional responses to catch trials, always consisting in the false perception of unilateral left touches. Furthermore, clear signs of USN were present in all tests (Table 2). EB was deeply unaware of her defective performance in extinction and neglect testing. Although she had a good awareness of her motor deficits, she generally attributed any other deficit of the daily life, i.e., neglecting objects on the left side, bumping into doorways or impaired reading abilities, to fatigue and impaired vision.

**Table 1:** **Testing for extinction**.

**Day of testing**	**Session**	**Left**	**Right**	**Bilateral**	**Catch**
1	First baseline	67	100	0	67
1	Delayed baseline	75	100	0	100
1	Endogenous attention shift	83	100	4	33
2	Pre-Hypnosis-1	83	100	0	100
2	Post-hypnosis-1	83	100	0	92
3	Pre-hypnosis-2	75	100	0	100
3	Post-hypnosys-2	100	100	33	100
3	Post-hypnosys-2 delayed	100	100	25	100

Percent of correct responses to unilateral and bilateral trials in the different testing sessions. Errors on bilateral trials always consisted of the omission of the left-sided stimulus. Errors on catch trials always consisted of the false report of a left-sided stimulation.

**Table 2:** **Neuropsychological evaluation of USN**.

**Session**	**Sentence reading (correct)**	**Figure and shape copying (score)**	**Star cancellation (left/right omissions)**	**Letter cancellation (left/right omissions)**	**Line bisection (score)**
First baseline	2^*^	1^*^	23/2^*^	26/6^*^	3^*^
Delayed baseline	3^*^	2^*^	20/1^*^	22/5^*^	3^*^
Endogenous attention shift	4^*^	2^*^	18/3^*^	19/6^*^	4^*^
Pre-hypnosis-1	3^*^	2^*^	19/0^*^	14/8^*^	6^*^
Post-hypnosis-1	4^*^	2^*^	18/0^*^	10/1^*^	6^*^
Pre-hypnosis-2	5^*^	1^*^	20/6^*^	12/6^*^	7
Post-hypnosis2	5^*^	2^*^	12/0^*^	14/2^*^	7

*Scores on the neuropsychological tests for USN in the different experimental conditions (see text for the scoring procedures and the asterisk indicates abnormal performance*.

### Effects of hypnosis on tactile extinction and USN (Tables 1, 2)

When tested for extinction (Table 1), EB showed a fluctuation in the performance on left unilateral trials across all sessions. On the other hand, there was a very clear and reliable pattern for the double bilateral stimulations: In the first two baseline sessions the left stimulus was never detected. Similarly, in the third session, when EB was instructed to orient her attention toward the left side, there was a non-significant increase of correct perception of bilateral trials (1/24 correct), but also an increase in false alarms (8/12) [CHI2(1) = 0,980, *p* = 0.3 and CHI2(1) = 2.5; *p* = 0.1, respectively]. This pattern suggests the adoption of an overall less conservative criterion in reporting left targets, following the attentional shift. Even the first hypnotic induction (Hypnosis-1) produced no effect on extinction. By contrast, after the second (specific) hypnotic session (Hypnosis-2) the perception of left touches on bilateral trials critically improved (8/24 stimuli correctly reported post-hypnosis vs. 0 pre-hypnosis, CHI2 (1) = 5,4; *p* < 0.05, with no difference in false alarms (100% correct) (Table 1). The improvement was still observed in the delayed post-hypnosis testing (6/24 stimuli correctly reported, CHI2 (1) = 4.5, *p* < 0.05. Also the response to left unilateral trials showed a marginally significant improvement in both the immediate and delayed post-hypnotic testing sessions (100% correct for both sessions vs. 9/12 correct before hypnosis, CHI2 (1) = 3.4, *p* = 0.06).

At odds with extinction, which showed remarkably stable results before the critical Hypnosis-2 session, the scores on most tests for USN showed a progressive improvement going from the first to the last repetition of the neuropsychological assessment (Table 2). More specifically, in the sentence reading test, some improvement was present in the baseline testing, in the unspecific hypnosis session, but not in the critical Hypnosis-2 session. In the figure and shape copying task, the amount of improvement in the critical Hypnosis-2 session was the same as in the baseline. The star cancellation was the only test showing some improvement in the Hypnosis-2 session, although some improvement was also found in the baseline test, while letter cancellation actually showed a worsening after hypnosis. Finally the line bisection showed no effect in the Hypnosis-2 session. Overall, the improvement found from the first baseline to the pre-Hypnosis-2 session was in most cases comparable, or superior, to that found following the critical Hypnosis-2 session and is likely due to unspecific learning effects, caused by the numerous repetitions of the testing battery. Due to this general pattern, and to the main target of the experimental procedure, which was extinction, USN scores were not further analyzed.

## Discussion

The present case shows that hypnotic suggestion can positively affect a post-stroke, chronic neuropsychological deficit, namely tactile extinction. Within the imaginative context based on the patient's spontaneous recalls of a pleasant situation during the hypnotic trance, it was suggested to EB to try and modulate (“play with,” as stated by the hypnotist) any somatosensory sensations coming from the right and left sides of the body, in the situation she was visualizing. In particular it was suggested to try and reduce the weight of right sided afferences, and increase that of left-sided ones. This suggestion was inspired by the theoretical notion that extinction consists of an ipsilesional bias in the competitive selection that the brain normally operates in the presence of multiple stimuli (Duncan, 1980, 1984; Bundesen, 1990). Due to this biased competition, even when stimuli are equally salient, the one presented ipsilesionally would be more likely perceived than the contralesional one (Driver et al., 1997). Accordingly, the suggestion of decreasing the weight of the ipsilesional stimulus and increasing that of the contralesional one was aimed at reducing stimulus competition, thus improving extinction. Such a specific content of the hypnotic verbalizations may also explain the selective effect of the procedure for tactile extinction, as compared to USN, in the critical Hypnosis-2 session.

The same increased contralesional processing would explain the improved perception of unilateral left stimuli, consistent with the idea that part of the contralesional somatosensory deficit occurring in right brain-damaged patients is not due to a primary sensory deficit but to impaired awareness (Sterzi et al., 1993).

Critically, hypnotic suggestion showed a stronger effect than the mere orienting of endogenous spatial attention towards the affected side without hypnosis. Indeed, as famously shown by Raz and colleagues (Raz et al., 2007; Raz and Campbell, 2011), hypnotic suggestion can strongly modulate attentional processes, including the performance on automatic attentional interference tasks, such as the Stroop effect, that would be typically refractory to any attentional effort made to override them. Our result is also in line with recent findings by Priftis et al. (2011) who showed that post-hypnotic suggestion can induce a neglect-like behavior in the response time to lateralized targets in healthy participants. Critically, in the same participants, the effect was much weaker following a mere condition of endogenous attentional orienting than in the case of post-hypnotic suggestion.

One main reason for the stronger effect of hypnotic suggestion over simple attention orienting in our patient could be that EB was unaware of her neuropsychological deficit, as typically observed in these patients (Vallar and Ronchi, 2006). This usually makes rehabilitation procedures based upon “bottom-up” or automatic mechanisms more effective than those based on explicit strategies that are typically aimed at increasing leftward attentional orienting in neglect and extinction (see Maravita et al., 2003; Fortis et al., 2010). In this view, hypnosis may represent an optimal strategy for the rehabilitation of deficits involving some degree of unawareness and anosognosia.

Noteworthy, the improvement of extinction found in EB occurred without the necessity for EB to adopt any overt strategy to overcome her sensory deficit, out of hypnosis. In order to explain how this was possible, it is useful to mention that experimental evidence showed that in patients with tactile extinction contralesional stimuli, although ignored during bilateral stimulations, can be nonetheless processed to some extent by the sensory systems, which can be completely unaffected by the lesion, even if the patient is not aware of them—an occurrence typically referred as implicit processing (Maravita, 1997; Berti et al., 1999). This evidence is in favor of the idea that somatosensory extinction corresponds to a deficit of higher level sensory processing, whereby sensory stimuli are processed only unconsciously, critically failing to reach the patient's awareness. In this view it is intriguing to think that any implicit information could be a precious anchor to try and improve the patient's performance. Through the critical verbalization given in trance of “playing” with the sensations coming from both hands, the patient was likely enabled to improve her awareness over residual tactile input coming from her left side, thus experiencing those inputs differently and opening the way to a better perception of left-sided stimuli even in the context of bilateral stimulations. This is unlikely to be due to increased endogenous attentional orienting toward the left side, given that an overt orienting of attention to the left was ineffective. One intriguing possibility is that the positive sensation imagined by the patient in the pleasant situation she visualized, may have acted as positive rewarding experience. Recent findings show that rewards can significantly bias attentional selection and learning (Della Libera and Chelazzi, 2006, 2009; Della Libera et al., 2011); in our case they could have increased the competitive weight of the contralesional stimulus, thus allowing them to reach awareness even during bilateral presentations.

It is important to say that the successful use of mainly indirect suggestions in the present case, which was given to the clinical experience of the hypnotist (MC) and the aforementioned theoretical issues, does not exclude that a more directive approach or an explicit post-hypnotic suggestion (e.g., suggesting to shift attention to the left) could be also successful. In fact, in the current literature, more directive hypnotic instructions have been typically used to bias behavior (see review in Oakley and Halligan, 2009). On one side it has to be said that, although the approach used in the present paper is not as directive as, for example, an overt suggestion of anesthesia (Rainville et al., 1997), or of attention modulation (Raz et al., 2007), it contains clear suggestions to guide the patient visual and sensory imagery, as well as suggestions to use these same imagined experiences in the daily life as a sort of healthy training exercise. In fact, the experimental approach used in the present work was not to use a “strict Ericksonian” approach (whose theoretical foundations are still a matter of discussion, Barber, 2000; Matthews, 2000; Peter and Revenstorf, 2000) in contrast with a “directive” one, although this approach is fruitfully used by many clinicians. By contrast, we think that indirect suggestions, post hypnotic commands and direct suggestions could be usefully compared, or even mixed, in the search of a successful rehabilitative approach.

Furthermore, although we think that using hypnosis in an imaginative context is surely a useful and promising approach, it remains possible that successful neuropsychological interventions in extinction patients may take advantage of visuomotor or haptic imagery, even without hypnotic suggestion (see related work on USN patients by Smania et al., 1997).

In order to assess the effectiveness of hypnosis, a critical question is clearly to assess the level of any “trance” state reached by the patient during the hypnosis session. In the present case we used an observational method, in which overt behavioral signs were taken as clues that a trance state was reached (see footnote 2). Of course, other variables could be taken into account, such as the level of hypnotic and non-hypnotic suggestibility of the patient (see discussion in Oakley and Halligan, 2011). In particular, the level of non-hypnotic suggestibility refers to the level of response to imaginative suggestions given out of hypnosis, while hypnotic suggestibility refers to the response to a suggestion while in hypnosis (Braffman and Kirsch, 1999). The level of non-hypnotic suggestibility has been found to correlate principally to response expectancy but also to other personality factors such as absorption, fantasy proneness and motivation (Braffman and Kirsch, 1999).

The results of the present paper are in line with growing evidence that hypnosis is a valuable tool to investigate neurocognitive functions, and with the recent suggestion that hypnosis could be used to improve different neuropsychological conditions such as USN (Oakley and Halligan, 2009; Priftis et al., 2011). To the best of our knowledge this work shows the first systematic evidence for the effect of hypnotic suggestion on a documented neuropsychological deficit in a brain-damaged patient. Although our experiment was aimed at testing any effect of hypnosis following a single experimental session and not as a long-lasting rehabilitative training, it opens the way to the possibility of a novel rehabilitative approach to neuropsychological disorders, in analogy to what already reported for other post-stroke deficits treated with hypnosis (Holroyd and Hill, 1989; Diamond et al., 2006). In particular, in future case studies a multiple-session approach could be used, which would favor the gradual implementation of specific suggestions tailored on the patient's needs and imaginative abilities, with the aim of training the patient to increase awareness of sensory events in order to obtain long-lasting improvements.

Noteworthy, in the present case, beside the specific improvement of extinction, the hypnotic procedure was effective in favoring attentional focusing and imagery in an otherwise emotional and distractible patient, as well as inducing deep muscular relaxation and reduction of spasticity in the plegic hand. It is therefore conceivable that hypnosis could exert beneficial effects not only by affecting the neuropsychological deficit and improving specific symptoms, but also by increasing the patient's compliance and motivation towards standard rehabilitation procedures targeting different sensory-motor functions.

### Conflict of interest statement

The authors declare that the research was conducted in the absence of any commercial or financial relationships that could be construed as a potential conflict of interest.

## Footnotes

^1^In the letter cancellation test (Diller and Weinberg, 1977) the patient was asked to mark all the “H” letters presented among other distracter letters and aligned in six horizontal lines. The score was the number of targets crossed out (53 on the left, and 51 on the right side of the sheet in total). The maximum difference between omission errors on the two sides of the sheet is two targets in neurologically unimpaired observers (Vallar et al., 1994b). In the star cancellation task the patient had to cancel star targets and ignore distracters (Wilson et al., 1987); the score was the number of correct targets crossed out (30 on the left, and 26 on the right-hand side in total). Ten comparable neurologically unimpaired participants tested in a previous experiment (Fortis et al., 2010) scored 0.5 omissions on average (SD 0.7, range 0–2), with the maximum difference between omission errors on the two sides of the sheet being one target. In the figure and shape copying test (Wilson et al., 1987), the score was given following a 0–4 range, based on the completeness of each drawn figure. The cut-off score for this test is 3. In the line bisection test the patient was requested to mark the midpoint of three 204-mm lines drawn on a single sheet of paper. The score range is 3–9, calculated on the amount of deviation from the objective center for each line, with a cut-off score of 7 (Wilson et al., 1987). In the sentence reading test patient was requested to read six sentences in Italian, consecutively presented in separate sheets of paper (score: 1 for each sentence read without omissions; score range: 0–6). Normal observers make no errors in this test (Pizzamiglio et al., 1990).

^2^The deepness of the hypnotic trance was systematically assessed using a custom-made questionnaire. The questionnaire is divided in two sections: the first comprises direct questions that are asked to the subject immediately after hypnosis to quantify his/her experience, the second is a check list in which a number of behavioural signs, observed in the participant during the trance state, are scored. Although this tool is not meant to score the hypnotic susceptibility but to qualify the actual experience of hypnosis, the scores at this questionnaire have been found to show some degree of correlation with the Stanford Hypnotic Susceptibility Scale (*r* = 0.47; *p* < 0.01) (data presented at the XI National Meeting of the “AMISI”—Italian Medical Association for the Study of Hypnosis-1998 Firenze “Quarant'anni di ipnosi in Italia:presente e futuro” G. Mosconi ed.). EB reached a score of 43% (a percent score relative to the maximum possible score) for the Hypnosis-1 and 46% for the Hypnosis-2 sessions. Such scores indicate that the patient reached a good, albeit not deep, hypnotic state.
